# Supplementary material for: Linker Histone H1.5 Contributes to Centromere Integrity in Human Cells
Source: bioRxiv. 2025 Jun 3:2025.06.03.657682. Preprint. [Version 1] doi: 10.1101/2025.06.03.657682 (PMC12191105; doi:10.1101/2025.06.03.657682)
Supplement: 1 [file NIHPP2025.06.03.657682V1-supplement-1.pdf]

bioRxiv preprint doi: https://doi.org/10.1101/2025.06.03.657682; this version posted June 3, 2025. The copyright holder for this preprint (which was not certified by peer review) is the author/funder. This article is a US Government work. It is not subject to copyright under 17 USC 105 and is also made available for use under a CC0 license.

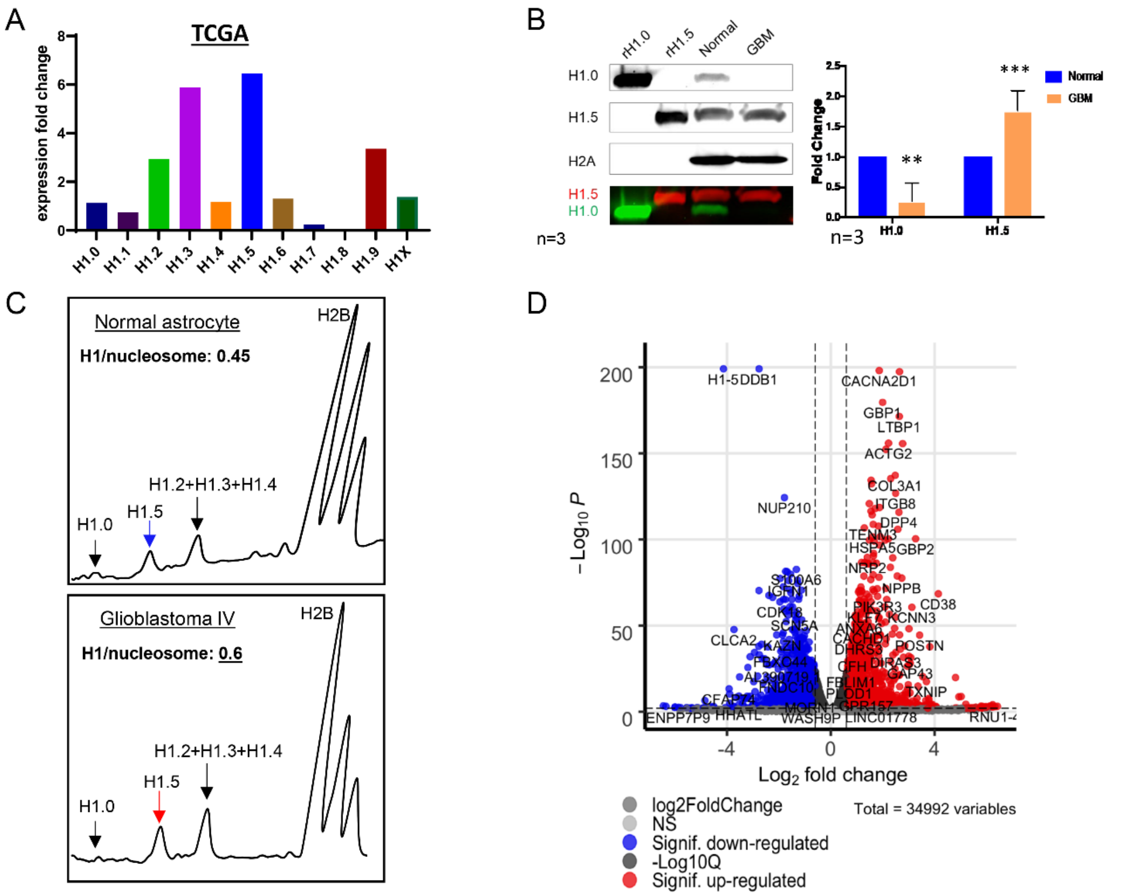

Figure S1. Histone H1.5 is a predominant H1 variant in astrocyte-lineage cells and regulates alpha-satellite transcription. (A) mRNA expression levels of histone H1 subtypes in Glioblastoma Multiforme (GBM) patient samples were analyzed using The Cancer Genome Atlas (TCGA) dataset. Among all variants, H1.5 shows the highest expression, suggesting a potential subtype bias in astrocytic tumors. (B) Western blot of hydroxylapatite-purified histone extracts from normal fetal astrocyte cells (SVGp12) and GBM cells (U138) confirm that H1.5 is elevated in GBM cells, accompanied by a notable reduction in H1.0. Quantification of band intensities (right) supports this subtype switch. Data are shown as mean ± standard deviation (SD) from n = 3 independent biological replicates. Statistical significance was determined using two-tailed Student’s t-test; *p < 0.05, **p < 0.01, ***p < 0.001, ****p < 0.0001. (C) High-performance liquid

bioRxiv preprint doi: https://doi.org/10.1101/2025.06.03.657682; this version posted June 3, 2025. The copyright holder for this preprint (which was not certified by peer review) is the author/funder. This article is a US Government work. It is not subject to copyright under 17 USC 105 and is also made available for use under a CC0 license.

chromatography (HPLC) analysis of acid-extracted histones reveals that H1.5 contributes significantly to the total H1 pool in glioblastoma cells compared to normal astrocytes. Peak identities are based on known elution profiles for individual H1 subtypes. (D) Volcano plot from RNA-seq analysis of SVGp12 cells treated with siScramble versus siH1.5 identifies significantly differentially expressed genes (FDR < 0.05). Alpha-satellite transcripts and multiple mitotic regulators are among the most downregulated genes upon H1.5 depletion.

bioRxiv preprint doi: https://doi.org/10.1101/2025.06.03.657682; this version posted June 3, 2025. The copyright holder for this preprint (which was not certified by peer review) is the author/funder. This article is a US Government work. It is not subject to copyright under 17 USC 105 and is also made available for use under a CC0 license.

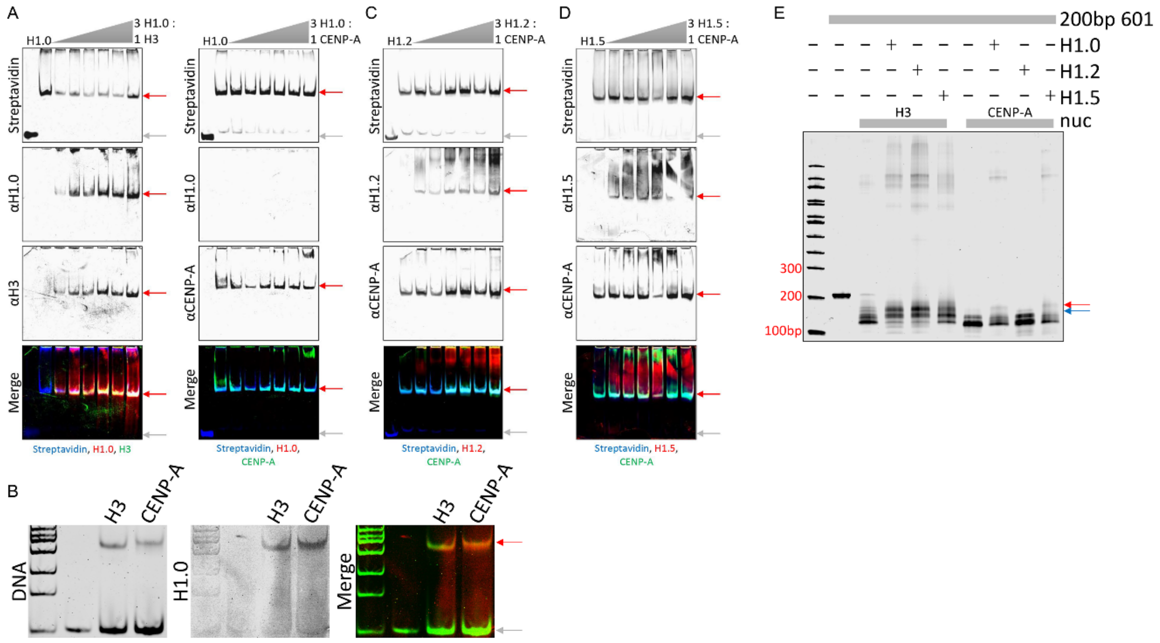

Figure S2. Histone H1s interact with H3 and CENP-A nucleosomes differently. (A) Histone H1.0 forms a stable nucleosome complex with H3- but not CENP-A- containing nucleosomes in the presence of 200 bp 601 positioning sequence with 5′ biotinylated ends. (B) H1.0 forms a stable nucleosome complex with both H3- and CENP-A- containing nucleosomes when using unmodified 200 bp 601 positioning sequence (no biotinylated ends). (C) H1.2 forms a stable nucleosome complex with CENP-A. (D) H1.5 forms a stable nucleosome complex with CENP-A. (E) Linker histones H1.0, H1.2, and H1.5 MNase protection signatures vary when bound to H3- or CENP-A nucleosomes.

bioRxiv preprint doi: https://doi.org/10.1101/2025.06.03.657682; this version posted June 3, 2025. The copyright holder for this preprint (which was not certified by peer review) is the author/funder. This article is a US Government work. It is not subject to copyright under 17 USC 105 and is also made available for use under a CC0 license.

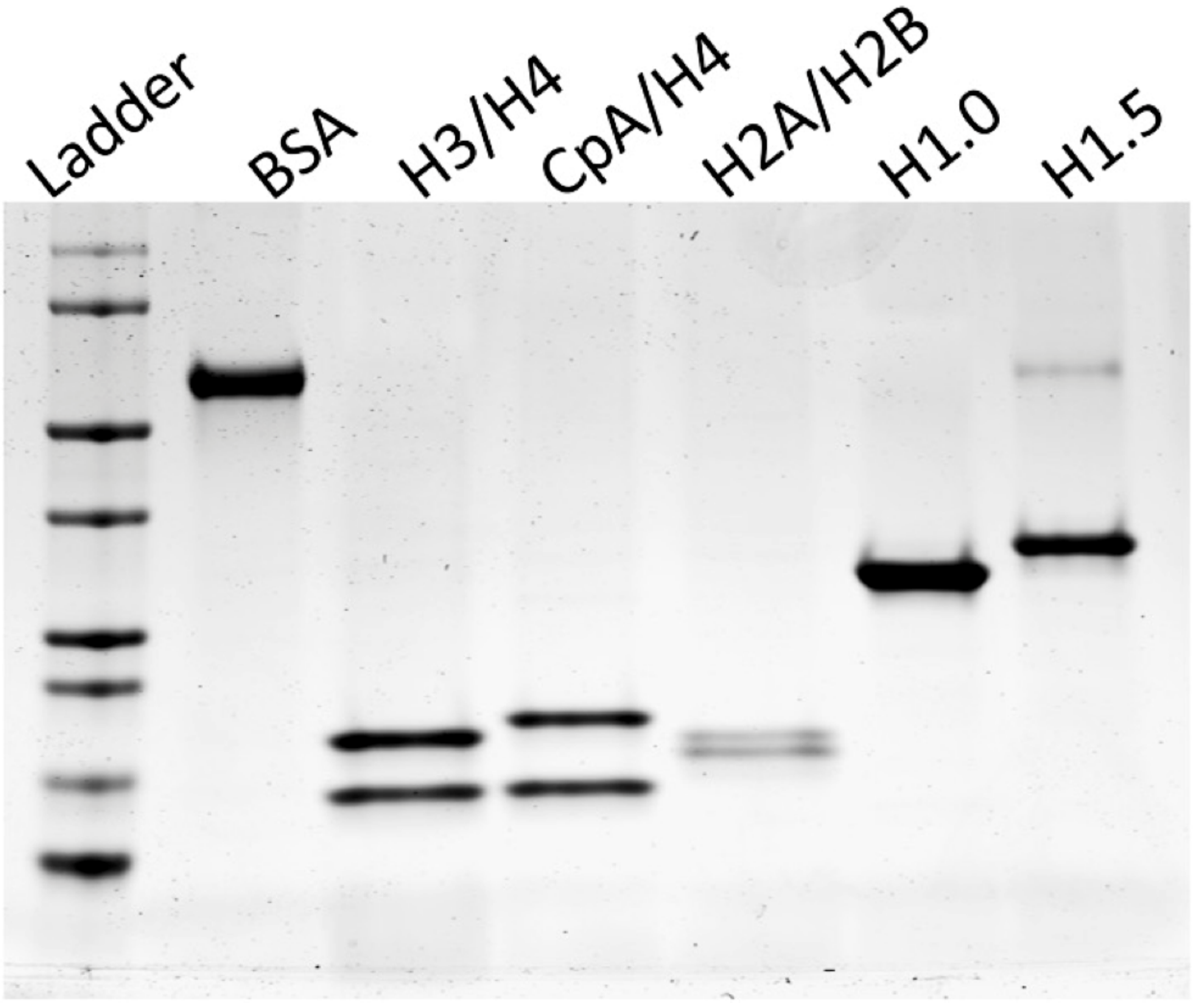

Figure S3. Protein validation for *in vitro* nucleosome reconstitution.

Coomassie-stained SDS-PAGE gel showing purified recombinant proteins used for nucleosome reconstitution: H3/H4 and CENP-A/H4 tetramers, H2A/H2B dimers, and linker histones H1.0 and H1.5.

bioRxiv preprint doi: https://doi.org/10.1101/2025.06.03.657682; this version posted June 3, 2025. The copyright holder for this preprint (which was not certified by peer review) is the author/funder. This article is a US Government work. It is not subject to copyright under 17 USC 105 and is also made available for use under a CC0 license.

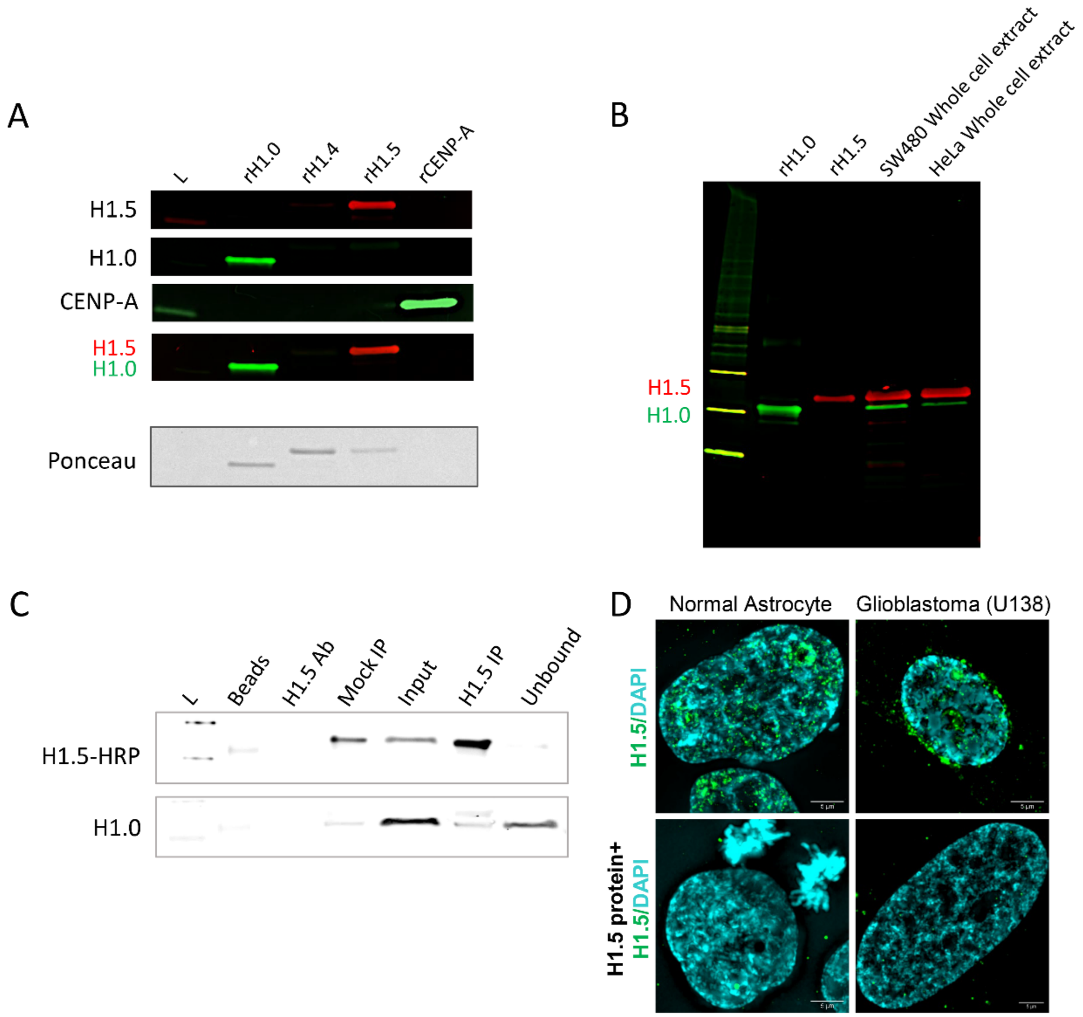

Figure S4. Validation of affinity-purified custom rabbit anti-H1.5 antibody for Western blotting, immunoprecipitation, and immunofluorescence. (A) Western blot showing antibody specificity against recombinant proteins H1.0, H1.4, H1.5, and CENP-A. The anti-H1.5 antibody detects only the H1.5 band (red), with no cross-reactivity to H1.0 or other tested proteins. Ponceau staining indicates equal loading. (B) Validation of H1.5 antibody specificity using histone extracts from colorectal and cervical cancer cell lines. Dual-color detection (H1.5 in red, H1.0 in green) confirms selective recognition of H1.5 across biological sources.

(C) Immunoprecipitation (IP) of endogenous H1.5 from cell lysates using the custom antibody.

bioRxiv preprint doi: https://doi.org/10.1101/2025.06.03.657682; this version posted June 3, 2025. The copyright holder for this preprint (which was not certified by peer review) is the author/funder. This article is a US Government work. It is not subject to copyright under 17 USC 105 and is also made available for use under a CC0 license.

Western blot probed with H1.5-HRP conjugate confirms efficient and specific pulldown of H1.5 compared to control IgG or H1.0 antibody IP. (D) Immunofluorescence validation of H1.5 signal in SVGp12 normal astrocytes and glioblastoma U138 cells. Pre-incubation of antibody with recombinant H1.5 protein (bottom panels) results in loss of nuclear signal, confirming specificity of detection. Nuclear morphology and H1.5 distribution differ between normal and cancer cells.

bioRxiv preprint doi: https://doi.org/10.1101/2025.06.03.657682; this version posted June 3, 2025. The copyright holder for this preprint (which was not certified by peer review) is the author/funder. This article is a US Government work. It is not subject to copyright under 17 USC 105 and is also made available for use under a CC0 license.

A

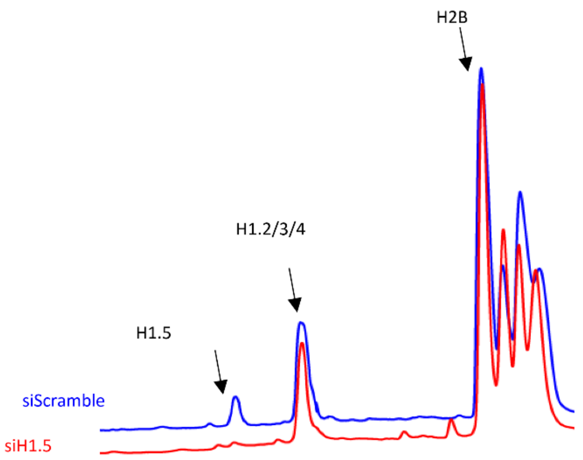

Sample	Average H1/nucleosome ratio (n=2)
**siScramble**	**0.44**
**siH1.5**	**0.29**

B

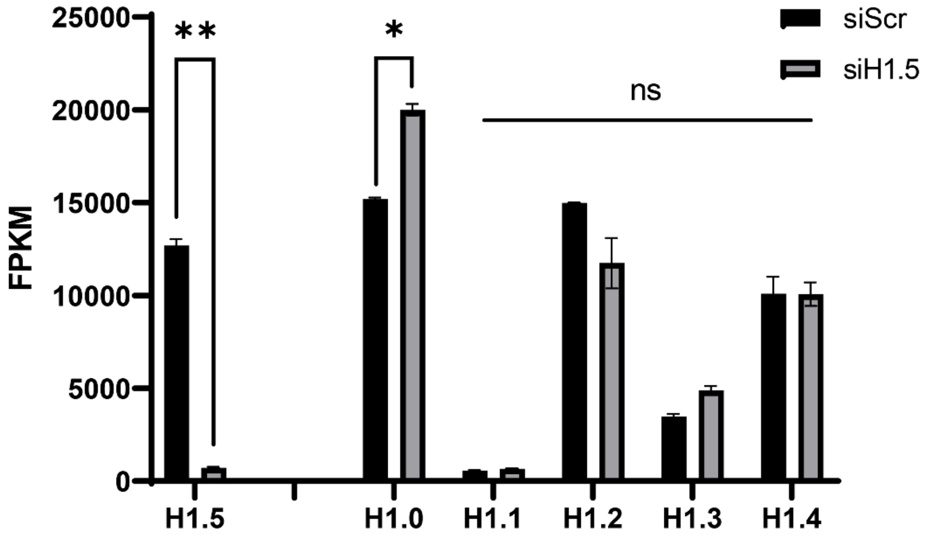

Figure S5. H1.5 is selectively reduced upon siRNA knockdown. (A) HPLC chromatograms of acid-extracted histones from siScramble (blue) and siH1.5-treated (red) SVGp12 cells show a selective reduction in H1.5 peak intensity following knockdown. Quantification of H1/nucleosome ratios (normalized to H2B) confirms a significant reduction of total H1 in siH1.5-

bioRxiv preprint doi: https://doi.org/10.1101/2025.06.03.657682; this version posted June 3, 2025. The copyright holder for this preprint (which was not certified by peer review) is the author/funder. This article is a US Government work. It is not subject to copyright under 17 USC 105 and is also made available for use under a CC0 license.

treated samples (0.29) compared to siScramble control (0.44). (B) RNA-seq quantification of histone H1 mRNA subtypes reveals a significant reduction in H1.5 ($p < 0.01$) and a mild but significant increase in H1.0 ($p < 0.05$) upon siH1.5 knockdown. No significant change (ns) was observed in H1.1, H1.2, H1.3, or H1.4 transcripts (n = 2 biological replicates, error bars indicate SEM).

bioRxiv preprint doi: https://doi.org/10.1101/2025.06.03.657682; this version posted June 3, 2025. The copyright holder for this preprint (which was not certified by peer review) is the author/funder. This article is a US Government work. It is not subject to copyright under 17 USC 105 and is also made available for use under a CC0 license.

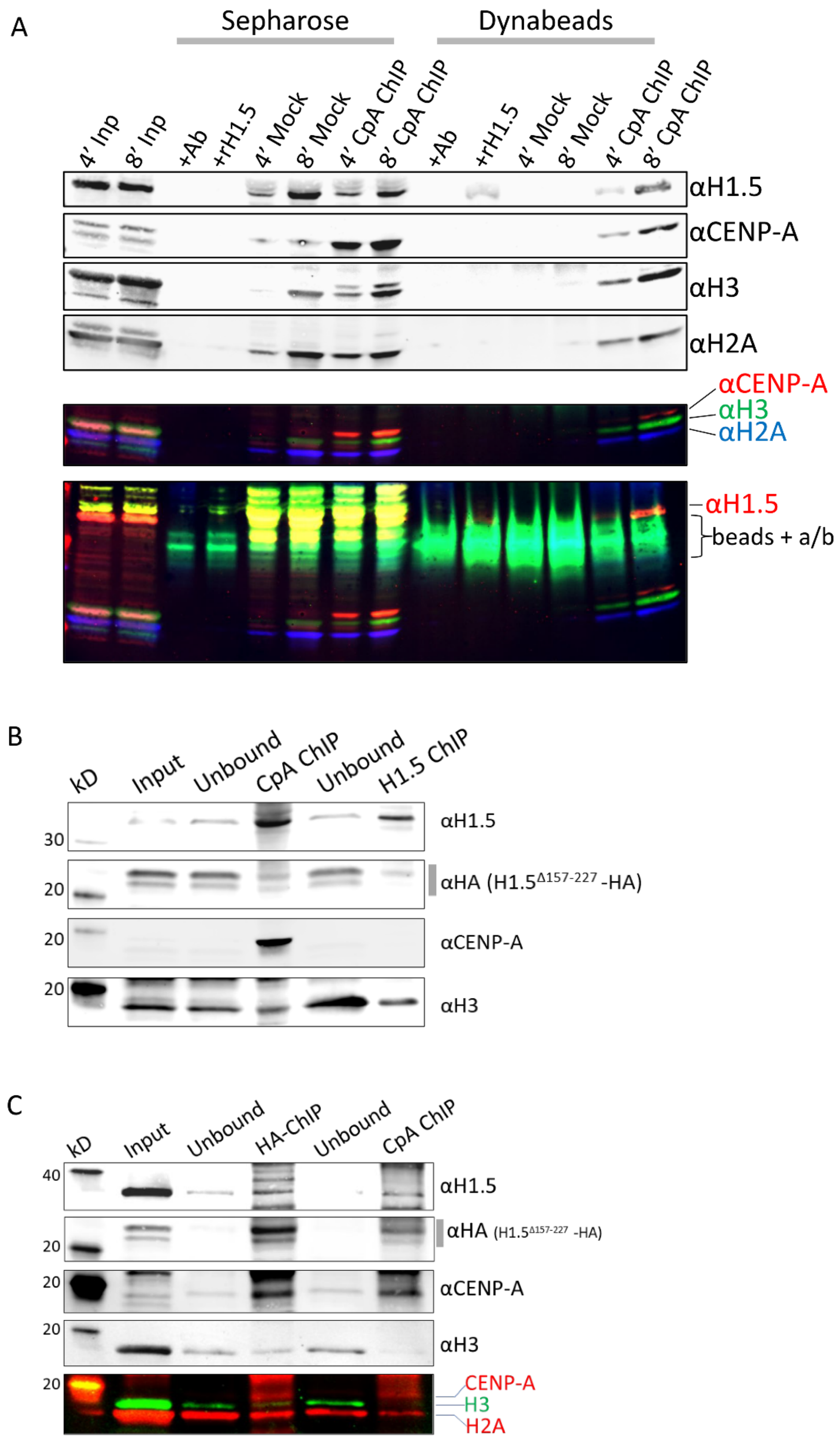

bioRxiv preprint doi: https://doi.org/10.1101/2025.06.03.657682; this version posted June 3, 2025. The copyright holder for this preprint (which was not certified by peer review) is the author/funder. This article is a US Government work. It is not subject to copyright under 17 USC 105 and is also made available for use under a CC0 license.

Figure S6. H1.5 associates with CENP-A nucleosomes in vivo. (A) In vivo ChIP verification of chromatin prepared using medium (4 min MNase digestion) or mononucleosome (8 min MNase digestion) conditions, using two bead formats: Sepharose and Dynabeads Protein A. Immunoprecipitation was performed with antibodies against H1.5, CENP-A, or control IgG. Western blotting reveals co-precipitation of H1.5 and CENP-A from chromatin, along with core histones H3 and H2A. Multiplex fluorescent detection of histone bands is shown below the Western panels. Notably, Dynabeads Protein A showed minimal non-specific binding in mock immunoprecipitated samples, and was thus used for all subsequent H1.5 ChIP experiments. (B) Co-IP using anti-CENP-A and anti-H1.5 antibodies from HeLa cells expressing wild-type or truncated H1.5 (Δ157–227-HA). CENP-A robustly co-precipitates with both endogenous and truncated H1.5, suggesting that the C-terminal tail is dispensable for the interaction. (C) Comparative Western blot analysis and fluorescent imaging of histone content in HA-ChIP and CENP-A-ChIP samples confirms the shared presence of CENP-A, H3, and H2A in nucleosome fractions pulled down with H1.5.

bioRxiv preprint doi: https://doi.org/10.1101/2025.06.03.657682; this version posted June 3, 2025. The copyright holder for this preprint (which was not certified by peer review) is the author/funder. This article is a US Government work. It is not subject to copyright under 17 USC 105 and is also made available for use under a CC0 license.

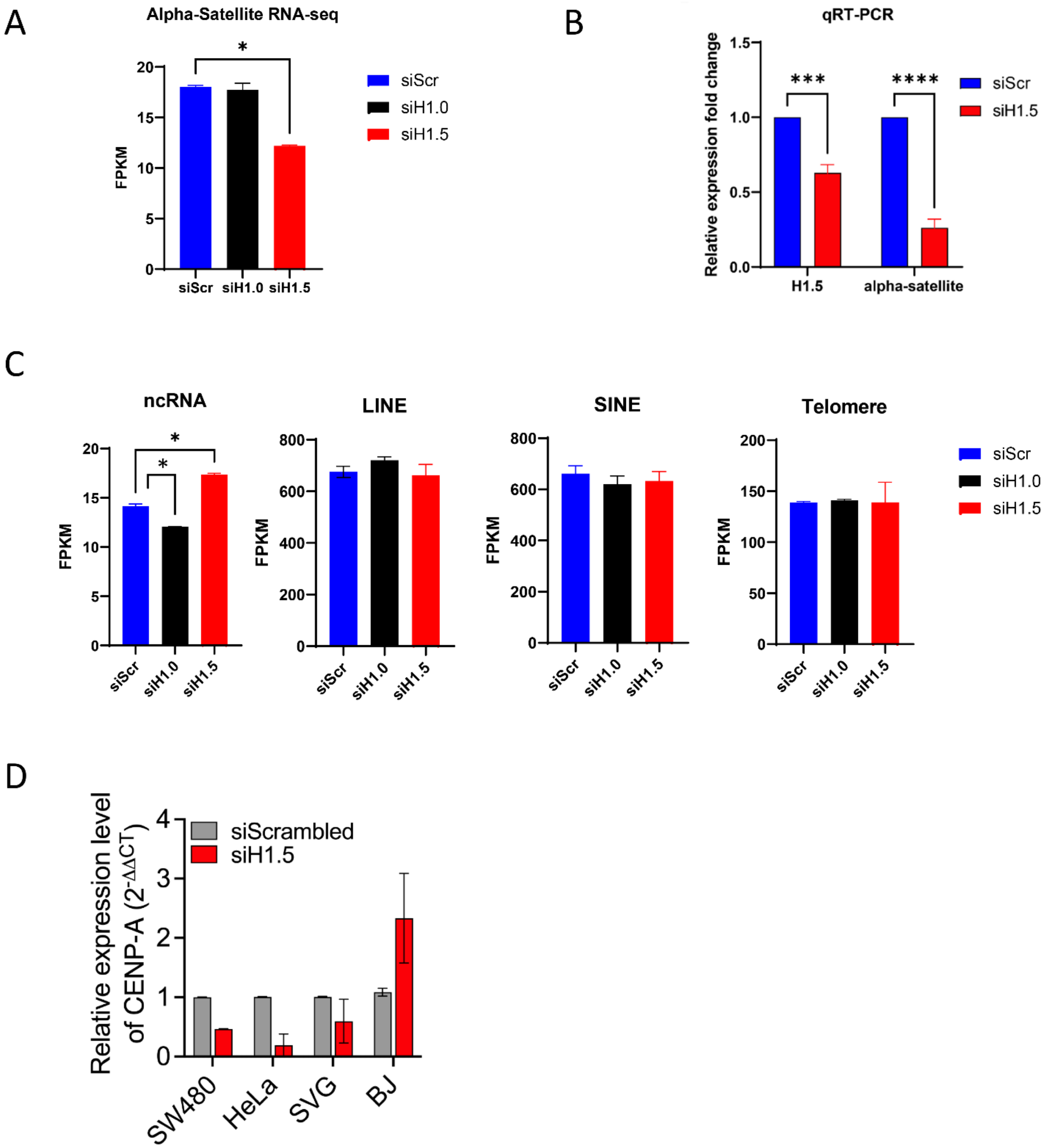

Figure S7. H1.5 depletion reduces α-satellite expression (A) RNA-seq quantification of α-satellite RNA in astrocytes treated with siScramble (blue), siH1.0 (black), or siH1.5 (red). H1.5 knockdown leads to a significant reduction in α-satellite expression, while H1.0 has no significant effect (unpaired two-tailed t-test, *$p < 0.05$) (B) qRT-PCR validation confirms effective

bioRxiv preprint doi: https://doi.org/10.1101/2025.06.03.657682; this version posted June 3, 2025. The copyright holder for this preprint (which was not certified by peer review) is the author/funder. This article is a US Government work. It is not subject to copyright under 17 USC 105 and is also made available for use under a CC0 license.

knockdown of H1.5 and associated reduction in alpha-satellite RNA in independent biological replicates. Expression levels are normalized to control housekeeping genes and presented as mean ± SEM ($p < 0.001$). (C) RNA-seq analysis of additional noncoding RNA categories, including total ncRNA, LINEs, SINEs, and telomeric repeats. Only the total ncRNA pool shows a significant reduction following H1.5 depletion (*$p < 0.05$). (D) CENP-A mRNA levels in SW480, HeLa, SVGp12, and BJ cells after siRNA-mediated knockdown of H1.5 (siH1.5, red) and compared them to siScramble control (gray) using quantitative RT-PCR. The relative expression values are based on calculations from the $2^{-\Delta\Delta Ct}$ method with normalization against GAPDH. The SW480, HeLa, and SVGp12 cells show decreased CENP-A transcripts when H1.5 levels decrease because centromere transcription becomes impaired. BJ fibroblasts demonstrate elevated CENP-A expression levels which indicate potential cell-type-specific differences in H1.5 function or the existence of compensatory mechanisms. The standard deviation is shown with error bars from three biological replicate measurements.

bioRxiv preprint doi: https://doi.org/10.1101/2025.06.03.657682; this version posted June 3, 2025. The copyright holder for this preprint (which was not certified by peer review) is the author/funder. This article is a US Government work. It is not subject to copyright under 17 USC 105 and is also made available for use under a CC0 license.

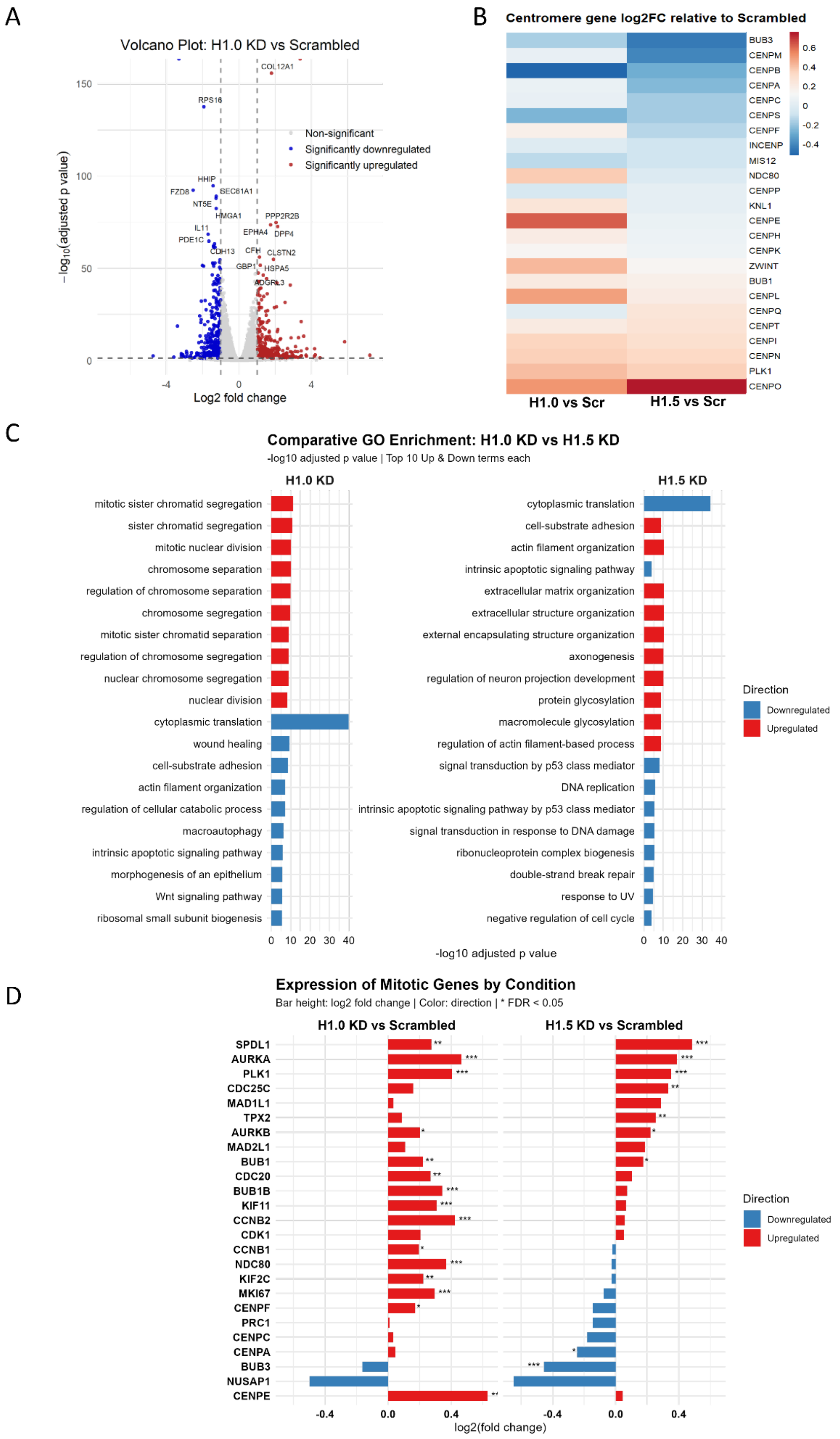

bioRxiv preprint doi: https://doi.org/10.1101/2025.06.03.657682; this version posted June 3, 2025. The copyright holder for this preprint (which was not certified by peer review) is the author/funder. This article is a US Government work. It is not subject to copyright under 17 USC 105 and is also made available for use under a CC0 license.

Figure S8. Comparative RNA-seq analysis of H1.0 and H1.5 depletion reveals distinct transcriptional effects. (A) Volcano plot showing differential gene expression upon H1.0 knockdown. Each point represents a gene, plotted by $\log_2$ fold change (x-axis) and $-\log_{10}$ p-value (y-axis). Genes with $p < 0.05$ and $\log_2$ fold change > 1 are shown in red (significantly upregulated), and those with $p < 0.05$ and $\log_2$ fold change < -1 are shown in blue, significantly downregulated). Non-significant genes are shown in light gray. Vertical dashed lines mark $\log_2$ fold change thresholds (± 1), and the horizontal dashed line indicates the p-value cutoff of 0.05. The top 10 most statistically significant up- and downregulated genes are labelled. (B) Heatmap showing log2 fold changes of curated centromere-associated genes between siH1.5 and siScramble conditions. (C) Comparative GO term enrichment analysis of the top 10 most significantly upregulated and downregulated biological processes following siH1.0 (left) or siH1.5 (right) knockdown relative to scramble control. Gene ontology enrichment was performed using clusterProfiler, with pathways ranked by adjusted p-value. (D) Targeted expression analysis of mitotic regulatory genes selected from the Reactome "M Phase" and "Mitotic Metaphase and Anaphase" gene sets (MSigDB). Bars represent $\log_2$ fold change for siH1.0 vs scramble (left) and siH1.5 vs scramble (right). Asterisks indicate statistical significance (* FDR < 0.05, ** FDR < 0.01, *** FDR < 0.001, **** FDR < 0.0001).

bioRxiv preprint doi: https://doi.org/10.1101/2025.06.03.657682; this version posted June 3, 2025. The copyright holder for this preprint (which was not certified by peer review) is the author/funder. This article is a US Government work. It is not subject to copyright under 17 USC 105 and is also made available for use under a CC0 license.

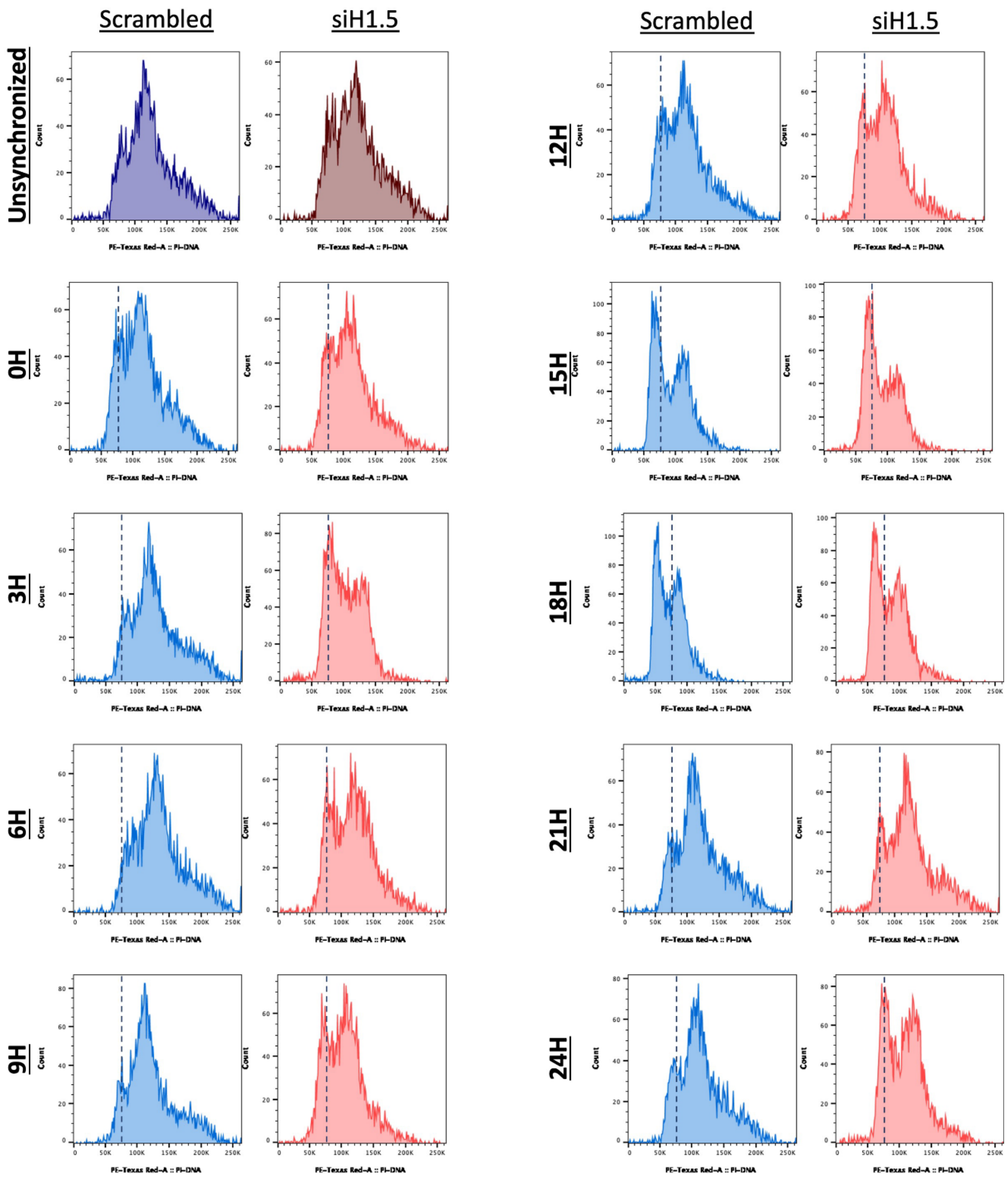

Figure S9. SVGp12 cells exhibit disrupted cell cycle progression after H1.5 knockdown. The representative flow cytometry (FACS) plots show propidium iodide–stained SVGp12 cells in two different conditions which include siScramble control (left panels in blue) and siH1.5-treated (right panels in red) during the cell cycle re-entry time course. A double thymidine block was

bioRxiv preprint doi: https://doi.org/10.1101/2025.06.03.657682; this version posted June 3, 2025. The copyright holder for this preprint (which was not certified by peer review) is the author/funder. This article is a US Government work. It is not subject to copyright under 17 USC 105 and is also made available for use under a CC0 license.

used to synchronize cells before transferring them into fresh media. Cell cycle dynamics were analysed by collecting samples at specified time intervals from 0 to 24 hours post-release to measure DNA content. The DNA content analysis of siScramble-treated cells demonstrates a synchronized progression through the cell cycle from G1 to S to G2/M phases before returning to G1 within 21–24 hours. Cells treated with siH1.5 experience delayed progression through the cell cycle with significant G2/M phase accumulation and expanded peaks beginning at 9 hours after release. The observed pattern reveals a compromised mitotic exit which can be explained by the triggering of the spindle assembly checkpoint or a delayed mitosis. The top row asynchronous samples serve as reference profiles for analysis. The data demonstrates that H1.5 plays an essential role in both timely mitotic progression and successful cell division completion in human glial SVGp12 cells.

bioRxiv preprint doi: https://doi.org/10.1101/2025.06.03.657682; this version posted June 3, 2025. The copyright holder for this preprint (which was not certified by peer review) is the author/funder. This article is a US Government work. It is not subject to copyright under 17 USC 105 and is also made available for use under a CC0 license.

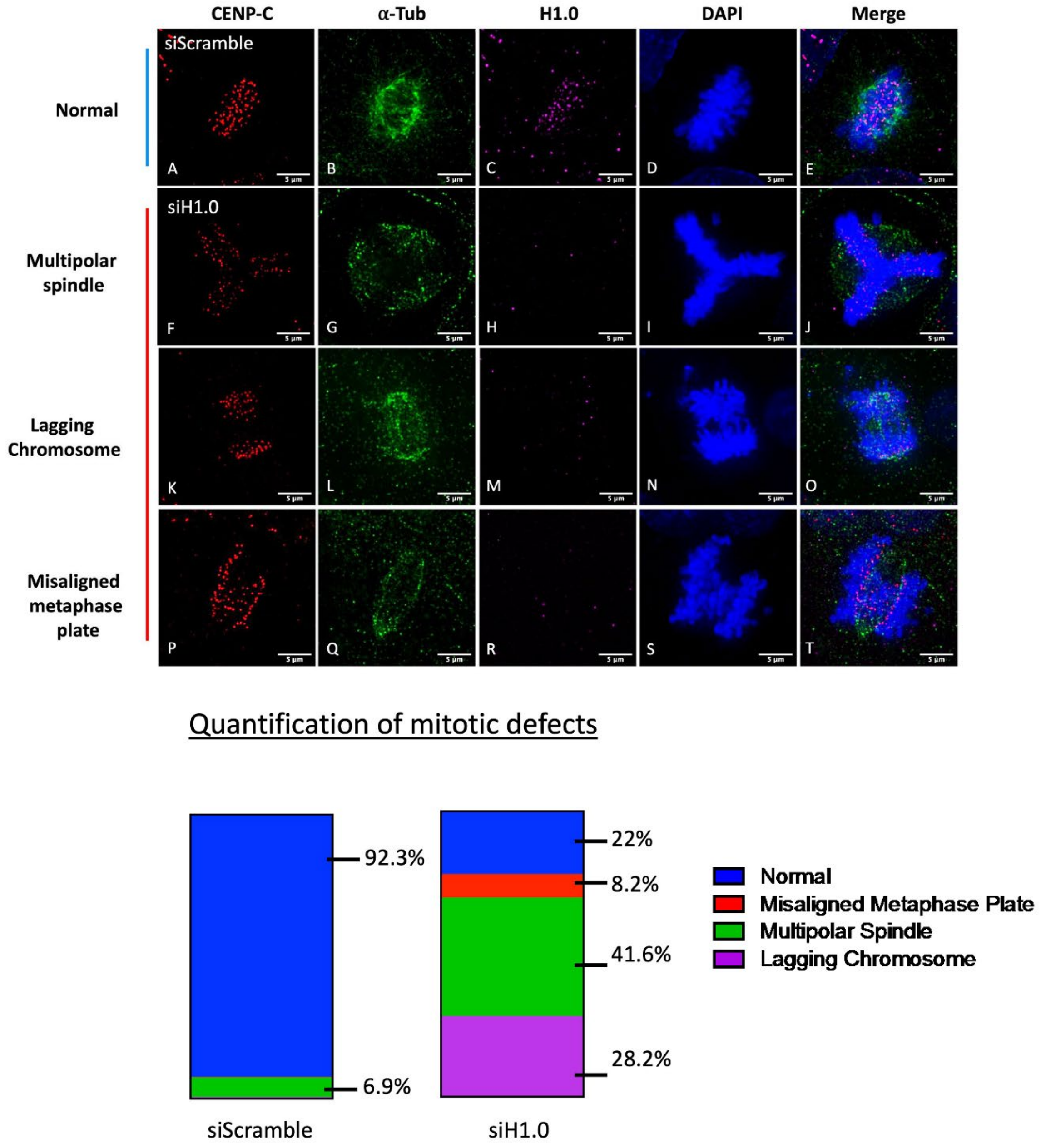

Figure S10. Human cells exhibit specific mitotic defects when histone H1.0 levels are reduced. SVGp12 cells transfected with either siScramble (top row) or siH1.0 (bottom three rows) show representative immunofluorescence images stained for CENP-C (red), α-tubulin (green), H1.0 (magenta), and DNA using DAPI (blue). siScramble control cells demonstrate typical mitotic progression with properly aligned metaphase plates and bipolar spindles. Cells with depleted

bioRxiv preprint doi: https://doi.org/10.1101/2025.06.03.657682; this version posted June 3, 2025. The copyright holder for this preprint (which was not certified by peer review) is the author/funder. This article is a US Government work. It is not subject to copyright under 17 USC 105 and is also made available for use under a CC0 license.

H1.0 levels show several mitotic abnormalities which manifest as multipolar spindles (G–J), lagging chromosomes (K–O), and misaligned metaphase plates (P–T). The reduction of H1.0 signal in knockdown cells demonstrates effective siRNA treatment. The combined and separate imaging channels demonstrate the spindle organization as well as the positions of chromosomes and the placement of histones. The bottom panel contains numerical data from 200 mitotic cells analysed under each experimental condition. Analysis of siScramble-treated cells reveals normal mitotic processes in 92.3% of cases while defects occur in 6.9%. Cells with siH1.0 knockdown show significant mitotic defects where 41.6% formed multipolar spindles while lagging chromosomes appeared in 28.2% of cells and 8.2% had misaligned metaphase plates. Untreated cells with siH1.0 show that only 22% complete mitosis without any visible defects.
